# Phenotype-driven assessment of the ancestral trajectory of sulfur biooxidation in the thermoacidophilic archaea Sulfolobaceae

**DOI:** 10.1128/mbio.01033-24

**Published:** 2024-07-02

**Authors:** Daniel J. Willard, Mohamad J. H. Manesh, Ryan G. Bing, Benjamin H. Alexander, Robert M. Kelly

**Affiliations:** 1 Department of Chemical and Biomolecular Engineering, North Carolina State University, Raleigh, North Carolina, USA; University of California Irvine, Irvine, California, USA

**Keywords:** archaea, sulfur, thermoacidophile, genomes, chemolithotrophy

## Abstract

Certain members of the family Sulfolobaceae represent the only archaea known to oxidize elemental sulfur, and their evolutionary history provides a framework to understand the development of chemolithotrophic growth by sulfur oxidation. Here, we evaluate the sulfur oxidation phenotype of Sulfolobaceae species and leverage comparative genomic and transcriptomic analysis to identify the key genes linked to sulfur oxidation. Metabolic engineering of the obligate heterotroph *Sulfolobus acidocaldarius* revealed that the known cytoplasmic components of sulfur oxidation alone are not sufficient to drive prolific sulfur oxidation. Imaging analysis showed that Sulfolobaceae species maintain proximity to the sulfur surface but do not necessarily contact the substrate directly. This indicates that a soluble form of sulfur must be transported to initiate cytoplasmic sulfur oxidation. Conservation patterns and transcriptomic response implicate an extracellular tetrathionate hydrolase and putative thiosulfate transporter in a newly proposed mechanism of sulfur acquisition in the Sulfolobaceae.

**IMPORTANCE:**

Sulfur is one of the most abundant elements on earth (2.9% by mass), so it makes sense that the earliest biology found a way to use sulfur to create and sustain life. However, beyond evolutionary significance, sulfur and the molecules it comprises have important technological significance, not only in chemicals such as sulfuric acid and in pyritic ores containing critical metals but also as a waste product from oil and gas production. The thermoacidophilic Sulfolobaceae are unique among the archaea as sulfur oxidizers. The trajectory for how sulfur biooxidation arose and evolved can be traced using experimental and bioinformatic analyses of the available genomic data set. Such analysis can also inform the process by which extracellular sulfur is acquired and transported by thermoacidophilic archaea, a phenomenon that is critical to these microorganisms but has yet to be elucidated.

## INTRODUCTION

Members of the thermoacidophilic archaeal family Sulfolobaceae (pH_opt_ <4; T_opt_ ≥ 65°C) have been identified throughout the globe in regions of volcanic activity, where they play a significant role in the acidification of these environments through the oxidation of reduced inorganic sulfur compounds (RISCs) (1). These archaea utilize inorganic substrates, including elemental sulfur and RISCs, as energy sources for chemolithotrophic growth (2). Among the archaea, only certain Sulfolobaceae exhibit this ability to oxidize sulfur and RISCs (3). Thus, discerning the genetic basis that distinguishes sulfur-oxidizing Sulfolobaceae from other members of the family (and, more broadly, the order Sulfolobales) can reveal the evolutionary changes that lead to a sulfur oxidation lifestyle. The first isolated member of this family, *Sulfolobus acidocaldarius*, was isolated in the presence of sulfur (4), and several strains of *S. acidocaldarius* were shown to oxidize elemental sulfur (5). However, the strain of *S. acidocaldarius* currently available through culture collections (strain 98/3) no longer has the capability to oxidize elemental sulfur, although its genome encodes enzymes attributed to this process (6).

Genetic tools in the Sulfolobales have been challenging to develop such that they are available only for the heterotrophic species *S. acidocaldarius* (7), *Sulfolobus islandicus* (8), and *Saccharolobus solfataricus* (9). As a result, approaches to understanding the mechanism of sulfur oxidation in the Sulfolobales have largely relied on the characterization of specific enzymatic functions (10–13). These approaches have identified several key steps in the cytoplasmic sulfur oxidation mechanism of the Sulfolobaceae, centered around an oxygen-dependent sulfur oxygenase reductase (SOR) that disproportionates elemental sulfur into sulfide and sulfite, forming thiosulfate through an abiotic side reaction (10). This central proposed role of SOR implies that oxygen must be present in order for sulfur oxidation to take place. Oxidation of sulfide and thiosulfate is then connected to the electron transport chain (ETC) through the membrane-bound sulfide:quinone oxidoreductase (SQR) (11) and thiosulfate:quinone oxidoreductase (TQO) (12). The product of TQO is tetrathionate, which is further processed in the cell by sulfur transport proteins DsrE3A and TusA (13), ultimately leading to NAD(P)^+^ reduction by the heterodisulfide reductase (HDR) complex (14). While some enzymatic activity of sulfite oxidation has been observed in *Acidianus ambivalens* (15), this activity has not been connected to a specific genetic sequence within the Sulfolobaceae. Ultimately, the fate of sulfite in the sulfur metabolism of the Sulfolobaceae is unknown.

While mechanisms for cytoplasmic sulfur oxidation have been proposed, several key pieces are missing. In particular, the extracellular acquisition of insoluble elemental sulfur has not been clearly elucidated. Elemental sulfur could be taken up by the cell by passive diffusion of nanoparticulate sulfur, the formation of which is facilitated by bisulfide (16) or by extracellular conversion to soluble RISCs, for which no clear enzymatic path has been identified. The only characterized extracellular enzyme from the Sulfolobaceae that is involved in RISC oxidation is a tetrathionate hydrolase (TetH) (17), but the role of this enzyme in the oxidation of elemental sulfur is unclear. Other studies have demonstrated that sequestering the organisms away from elemental sulfur prevents sulfur oxidation from taking place, suggesting that direct cell-substrate interaction, or at least proximity to elemental sulfur, is necessary to facilitate sulfur acquisition (16).

Even with a long-standing interest in the Sulfolobaceae since the initial discovery of *S. acidocaldarius* (4), the pan-genome of the family remains open (18), and new species continue to be isolated and sequenced (19, 20). The ever-expanding collection of sequenced genomes in the Sulfolobaceae provides a powerful tool for understanding their mode of sulfur oxidation more deeply. To date, differential gene and protein expression analysis of sulfur oxidation has focused on individual organisms (6, 21) rather than looking for common threads throughout the Sulfolobaceae. Conversely, direct comparison of genetic content in the Sulfolobales tends to be largely computational and targets the broader metabolism of these organisms (18, 22), rather than targeting a specific metabolic function. Here, the aim was to connect phenotypic evidence for sulfur oxidation capabilities across the Sulfolobaceae with comparative genomic and transcriptomic analysis to further decipher the details of sulfur oxidation and to elucidate novel features of the mechanism for the acquisition and oxidation of RISCs.

## RESULTS

### Benchmarking-engineered sulfur oxidation performance

**Fig 1:**
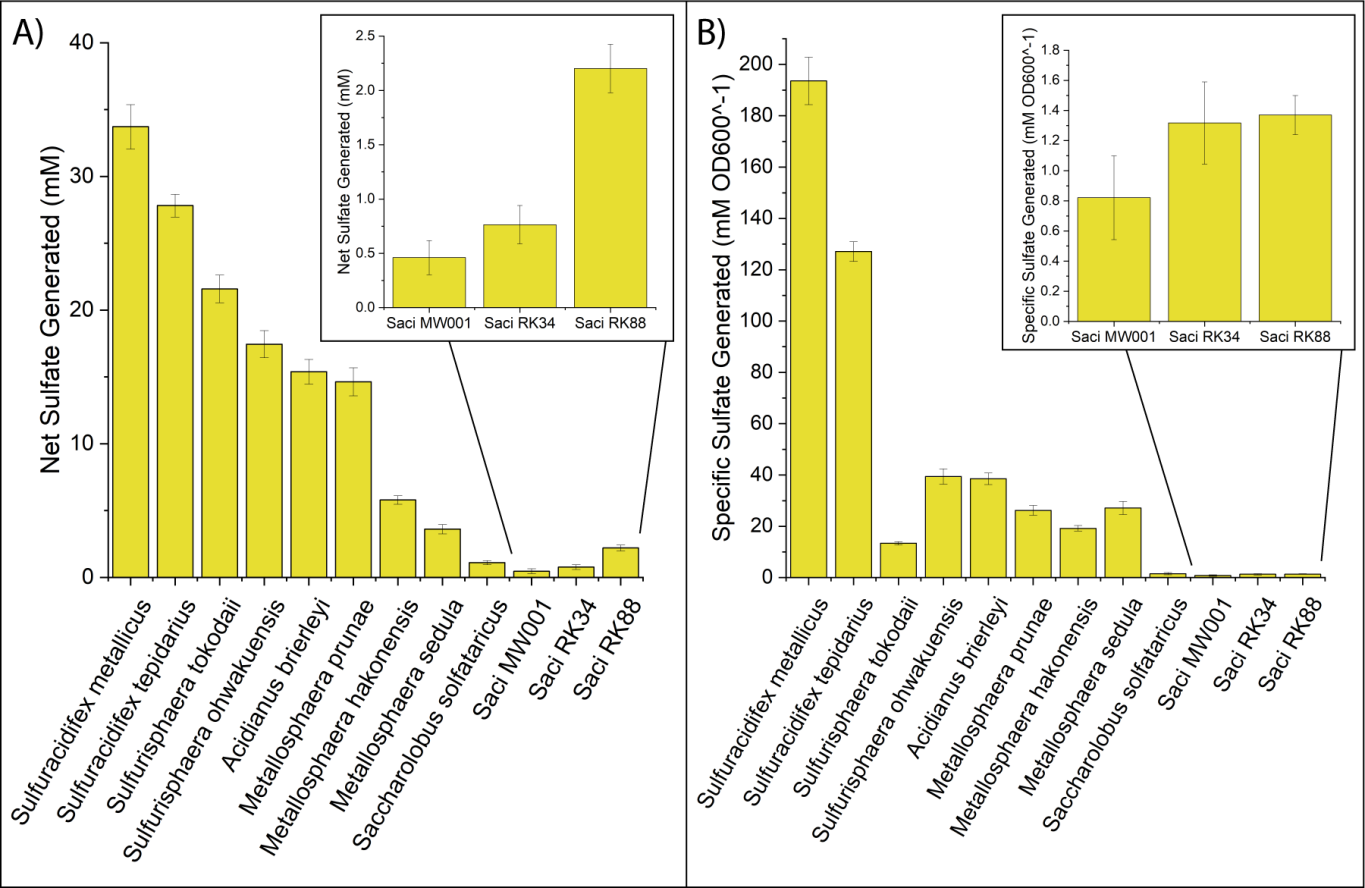
Mean sulfate generation (**A**) and specific sulfate generation scaled by cell density (**B**) of 72 h cultures for 10 Sulfolobaceae species and two engineered strains of *S. acidocaldarius*. Insets show a zoomed-in view of the sulfate generation values for the engineered *S. acidocaldarius* strains. Error bars indicate one SD using three biological replicates.

**Fig 2:**
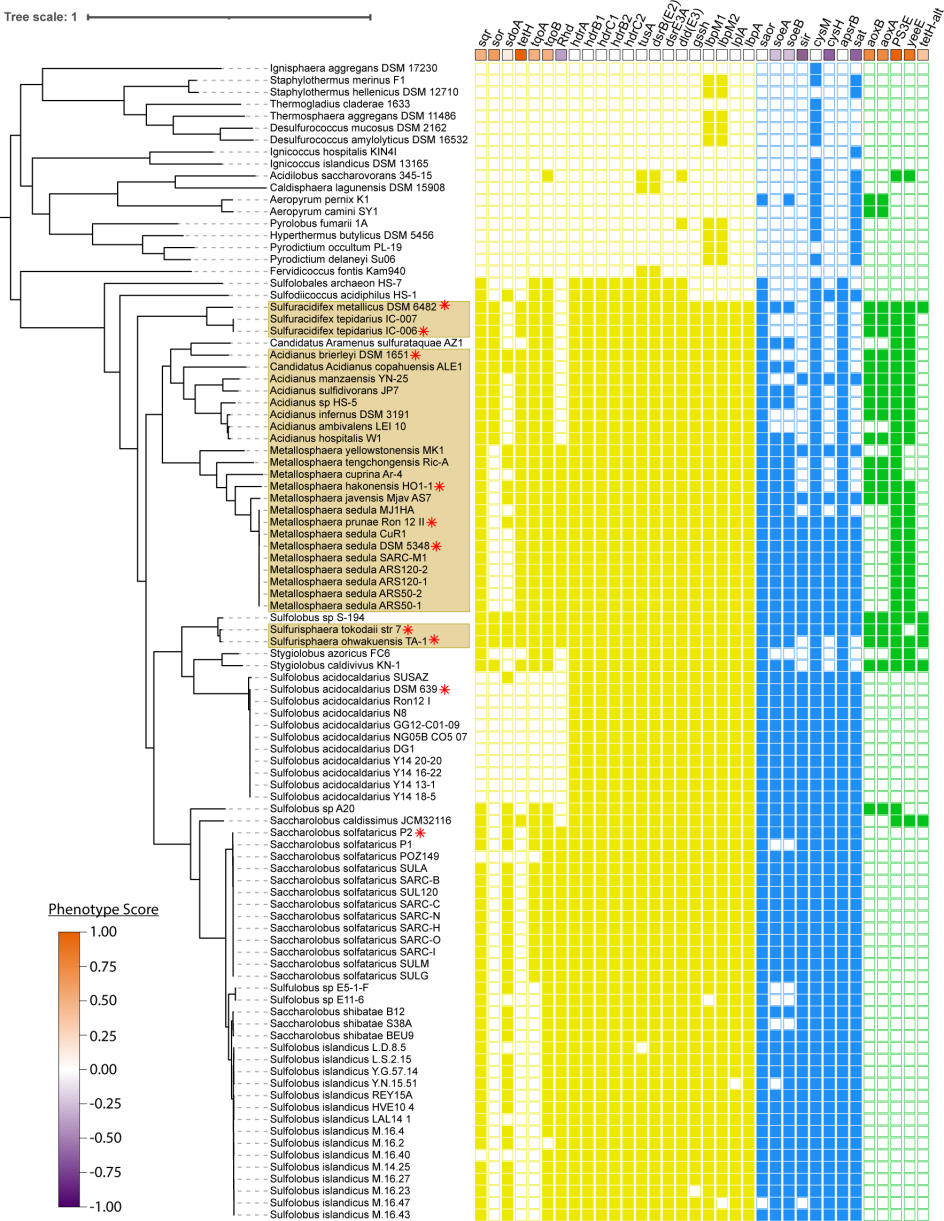
Phenotype scores of known and proposed sulfur metabolism genes. Genes are split into sulfur oxidation (yellow), sulfate assimilation/sulfite oxidation (blue), and potential novel sulfur genes (green). Species evaluated for sulfur oxidation phenotype in this work are indicated by a red asterisk. Phenotype scores are calculated by the presence/absence of each gene in these indicated species, with a score of 1.00 (orange) indicating the presence only in prolific sulfur oxidizers and a score of −1.00 (purple) indicating the presence only in basal sulfur oxidizers.


Ten Sulfolobaceae species were evaluated for sulfate production from elemental sulfur after a culturing period of 72 h. Of the 10 species, the two *Sulfuracidifex* species generated the most sulfate, both in terms of net production (Fig. 1A) and when normalized to planktonic cell density (Fig. 1B); thus, these two species were designated as “strong” sulfur oxidizers. In contrast, both *S. solfataricus* and *S. acidocaldarius* MW001 generated 1 mM or less of sulfate during that same period, rendering them as “weak” sulfur oxidizers. The remaining six Sulfolobaceae evaluated produced between 3.6 and 21.6 mM of sulfate over the 72-h period. These were characterized as “moderate” sulfur oxidizers, with the exception of *Sulfurisphaera tokodaii*; despite generating 21.6 mM sulfate, the sulfate production normalized to cell density was lower than all species tested besides *S. solfataricus* and *S. acidocaldarius* MW001. Therefore, the high total sulfate concentration was attributed to significant cell growth rather than particularly efficient sulfur oxidation, and *S. tokodaii* was considered to be somewhere between a “weak” sulfur oxidizer and a “moderate” sulfur oxidizer. Using these phenotypic groupings, a pangenome matrix of the 10 Sulfolobaceae was parsed for a correlation between gene conservation and sulfur oxidation capabilities (Fig. 2). Homologous gene clusters were categorized by a phenotype score according to Equation 1,

[1] $$Score_{Phe}=\frac{N_{A,gene}}{N_{A,species}}-\frac{N_{B,gene}}{N_{B,species}}$$

where the “A” group refers to the Sulfolobaceae species that demonstrated more than weak sulfur oxidation, the “B” group refers to the Sulfolobaceae species that demonstrated weak sulfur oxidation, N_i,gene_ is the number of homologs for a particular gene cluster in each group, and N_i,species_ is the number of species in each group. Phenotype scores can, therefore, range from −1, indicating a complete inverse correlation between that gene’s presence and a species’ ability to oxidize sulfur, to 1, indicating a direct correlation of the same. Notably, none of the cytoplasmic sulfur oxidation genes had a phenotype score above 0.625, indicating that these genes are not necessarily markers of strong sulfur oxidation. The only sulfur metabolism gene with a phenotype score of 1, indicating a complete correlation with the sulfur oxidation phenotype groupings, was the *tetH* gene, encoding an extracellular TetH (17), which does not yet have a definitive role in elemental sulfur oxidation.

In addition to the 10 Sulfolobales species assessed, two engineered strains of *S. acidocaldarius* MW001 were also evaluated for sulfate production: one strain containing the *sor* and *tqoAB* genes from *S. tokodaii* (*S. acidocaldarius* RK34) and one strain containing the *sor*, *tqoAB*, and *sqr* genes from *S. tokodaii* (*S. acidocaldarius* RK88). Thus, the RK88 strain encodes homologs to all of the previously characterized cytoplasmic enzymes involved in archaeal sulfur oxidation. Relative to the *S. acidocaldarius* MW001 parent strain, the RK34 and RK88 strains showed modest increases in sulfate production (Fig. 1A and B insets). The specific sulfate production of the RK88 strain was comparable to the RK34 strain, but the total sulfate production of RK88 was much improved, indicating that the RK88 strain can reach higher cell densities while oxidizing sulfur compared to its parent strain. However, the increased sulfate production in either of the engineered *S. acidocaldarius* strains failed to reach the sulfate levels seen in any of the strong or moderate sulfur oxidizers. In fact, the sulfate production from the RK88 strain is comparable to *S. solfataricus* P2, an obligate heterotroph that contains all of the sulfur genes introduced to the RK88 strain except *sor*. Thus, the known cytoplasmic components of sulfur oxidation alone are insufficient to impart strong sulfur oxidation to an engineered strain of *S. acidocaldarius*.

### Evaluating surface interaction with elemental sulfur

**Fig 3:**
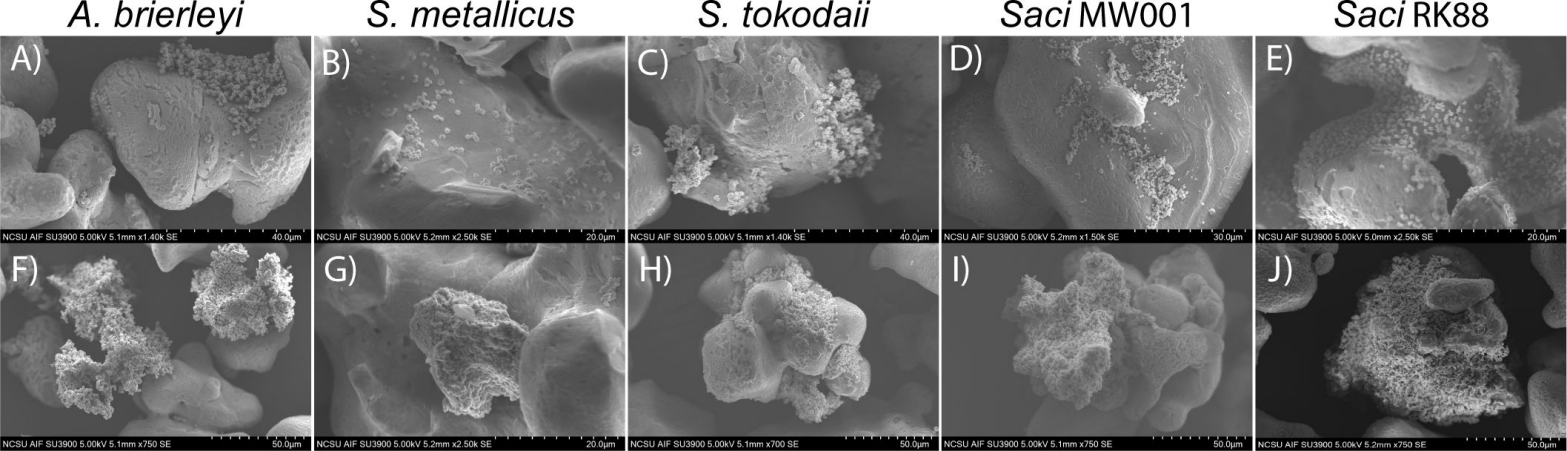
SEM images of Sulfolobaceae cultures attached to elemental sulfur: *Acidianus brierleyi* (**A and F**); *Sulfuracidifex metallicus* (**B and G**); *S. tokodaii* (**C and H**); *S. acidocaldarius* MW001 (**D and I**); *S. acidocaldarius* RK88 (**E and J**). Stages of cluster formation in *A. brierleyi* showing initial cell attachment (**K**), cell propagation at the sulfur surface (**L**), and formation of cell aggregates tethered to the sulfur surface (**M**).

A possible explanation for the relatively low sulfate oxidation capabilities of the RK88 strain is the requirement of surface interaction with the sulfur substrate. Scanning electron microscopy (SEM) imaging of *S. acidocaldarius* MW001 and RK88 strains revealed cells attached to the elemental sulfur in both cases (Fig. 3D, E, I and J). Cells were distributed across the sulfur surface and directly in contact with sulfur, indicating that cell proximity to the sulfur substrate was not a factor in the RK88 strain’s limited ability to oxidize sulfur.

**Fig 4:**
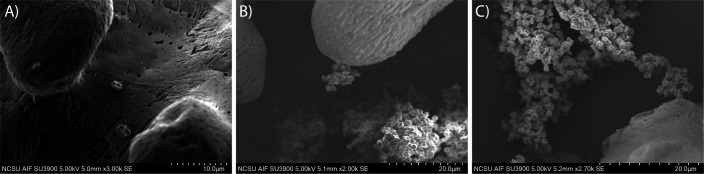
Stages of cell cluster formation in *A. brierleyi* attached to elemental sulfur. *A. brierleyi* cells associate with the surface initially as a single cell (**A**), propagate at the sulfur surface (**B**), and form large cell aggregates tethered to the sulfur surface (**C**).

As a point of reference, cultures of *Acidianus brierleyi*, *Sulfuracidifex metallicus*, and *S. tokodaii* were also imaged to assess cell-substrate interaction (Fig. 3A, B, C, F, G and H). Interestingly, these species did not spread out across the sulfur substrate in the manner of the *S. acidocaldarius* strains. Instead, they formed large aggregates tethered to the surface of the sulfur substrate through only a small fraction of cells. In the case of *A. brierleyi*, cells were observed in what appears to be multiple stages of this aggregate formation, propagating from a single cell attached to the sulfur surface (Fig. 4A) into a cluster of cells near the sulfur surface (Fig. 4B) and ultimately extending out and away from the sulfur surface (Fig. 4C). This indicates that the proximity of the cells to the sulfur substrate, rather than direct cell-to-sulfur contact, is sufficient for sulfur oxidation.

### Transcriptomic response of sulfur metabolism genes

The low sulfate production of the RK88 strain was not attributed to a lack of physical association with the sulfur substrate. Therefore, the genetic components of sulfur oxidation were explored further in *A. brierleyi*, *Sulfurisphaera ohwakuensis*, and *S. tokodaii* through transcriptomic response to elemental sulfur. This was also assessed for *S. acidocaldarius* MW001 to check for patterns that correlated with sulfur oxidation capabilities.

**Fig 5:**
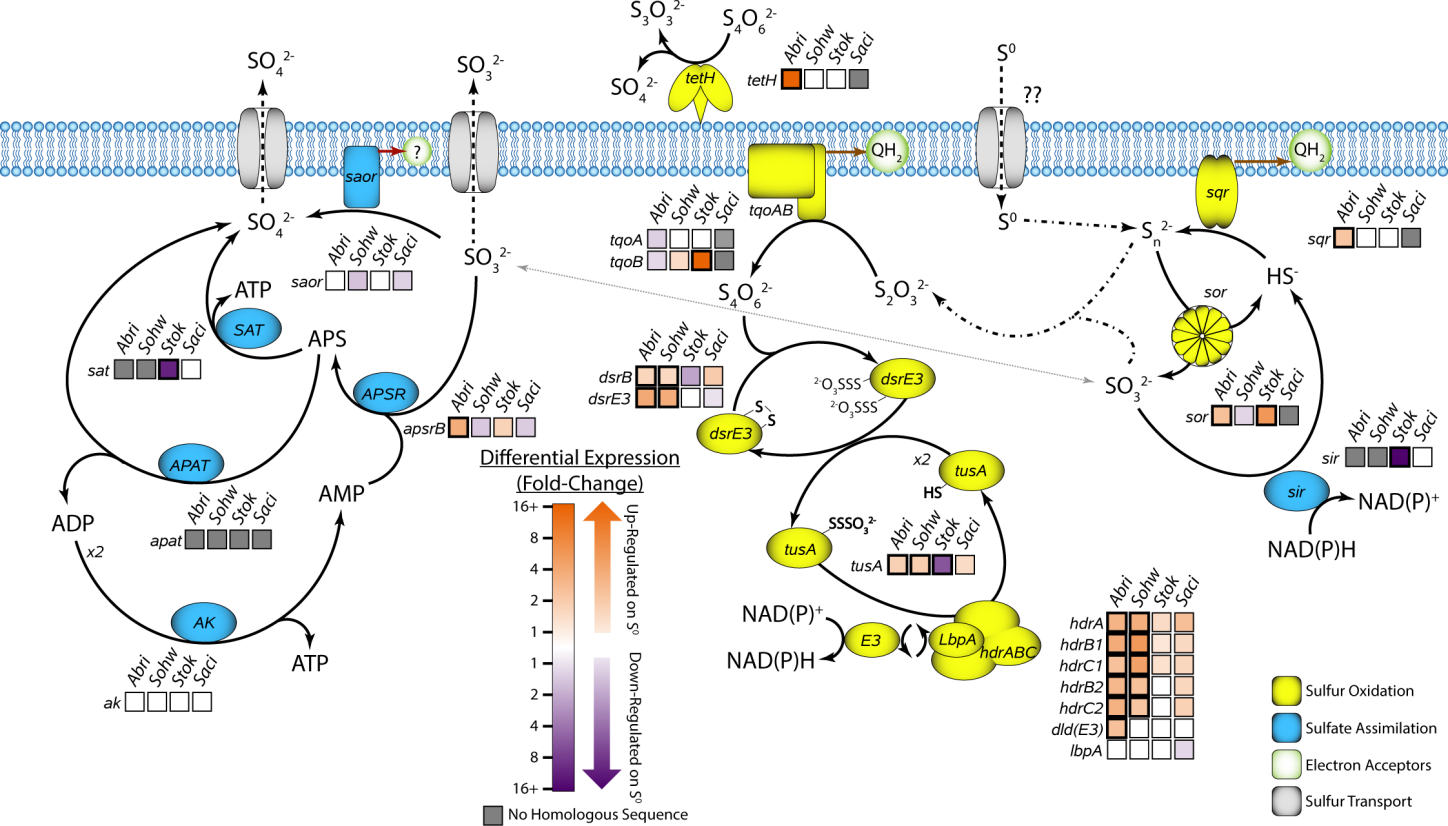
Transcriptomic response of known sulfur metabolism genes from *A. brierleyi*, *S. ohwakuensis*, *S. tokodaii*, and *S. acidocaldarius*. Genes upregulated in the presence of elemental sulfur are shown in orange, and genes downregulated in the presence of elemental sulfur are shown in purple. Gray boxes indicate that the gene is absent in the particular organism. Bold outlines indicate the differential expression is considered statistically significant by F-test. Enzymes represented in yellow are associated with sulfur oxidation, and enzymes represented in blue are associated with sulfate assimilation or sulfite oxidation. *Abbreviations: sqr* (sulfide:quinone oxidoreductase), *sir* (sulfite reductase), *sor* (sulfur oxygenase reductase), *hdrA*/*hdrB1*/*hdrC1*/*hdrB2*/*hdrC2* (heterodisulfide reductase complex), *dld(E3)* (dihydrolipoamide dehydrogenase), *lbpA* (lipoate binding protein), *dsrB*/*dsrE3* (disulfide reductase), *tusA* (sulfur carrier protein), *tqoA*/*tqoB* (thiosulfate:quinone oxidoreductase), *tetH* (tetrathionate hydrolase), *saor* (sulfite:acceptor oxidoreductase), *apsrB* (adenylylsulfate reductase), *apat* (adenylylsulfate:phosphate adenyltransferase), *sat* (sulfate adenylyltransferase), and *ak* (adenylate kinase).

The transcriptomic response of genes known to be involved in sulfur oxidation varied among the four species (Fig. 5). The highly conserved genes encoding the HDR complex were the only sulfur oxidation genes consistently upregulated in all species. *A. brierleyi* exhibited significant upregulation of the *sqr*, *tetH*, and *sor* genes while downregulating the genes encoding TQO. The two *Sulfurisphaera* species exhibited conflicting profiles of sulfur oxidation genes, with *S. ohwakuensis* upregulating the *sqr*, *dsrE3A*, and *tusA* genes and one subunit of TQO and *S. tokodaii* upregulating only the *sor* gene and a TQO subunit. While a transcriptomic response for the *tetH* gene from *S. ohwakuensis* was not detected, a homologous protein, annotated as a “pyrrolo quinoline quinone(PQQ)-binding-like beta-propeller protein,” was upregulated nearly 11-fold on sulfur. This homologous protein is also predicted to have a signal peptide and could serve a similar function in *S. ohwakuensis* to the TetH protein conserved in other species. Genetic components of sulfite oxidation/sulfate assimilation were largely unresponsive to the presence of sulfur.

### Novel genes linked to sulfur oxidation

**Fig 6:**
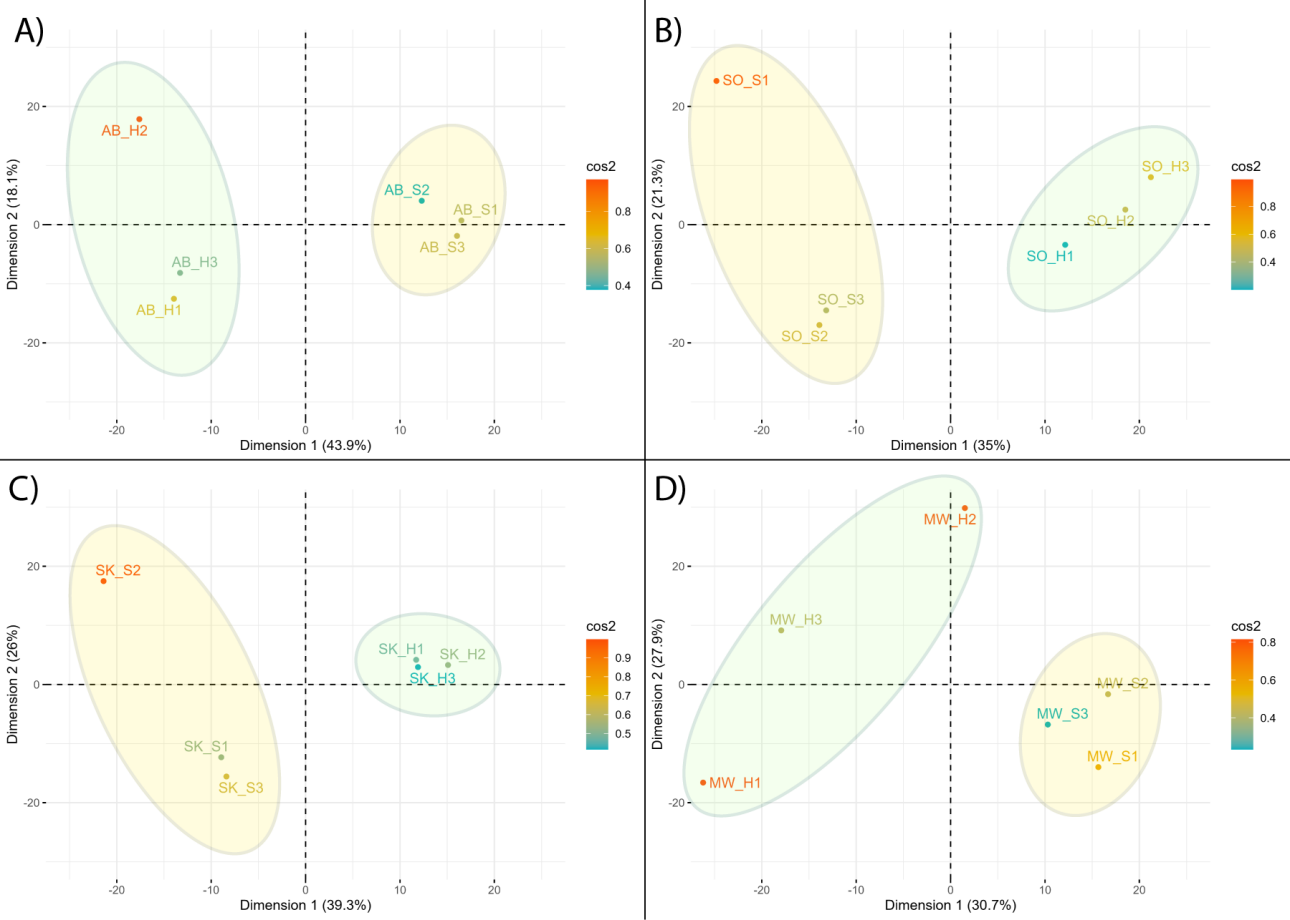
PCA of differential gene expression in response to elemental sulfur presence for biological replicates of *A. brierleyi* (**A**), *S. ohwakuensis* (**B**), *S. tokodaii* (**C**), and *S. acidocaldarius* MW001 (**D**). Dimension 1 of the PCA primarily describes the variation related to the presence of elemental sulfur. All conditions were run in biological triplicate; cultures grown without sulfur present are contained in a green circle, while cultures grown with sulfur present are contained in a yellow circle.

**Fig 7:**
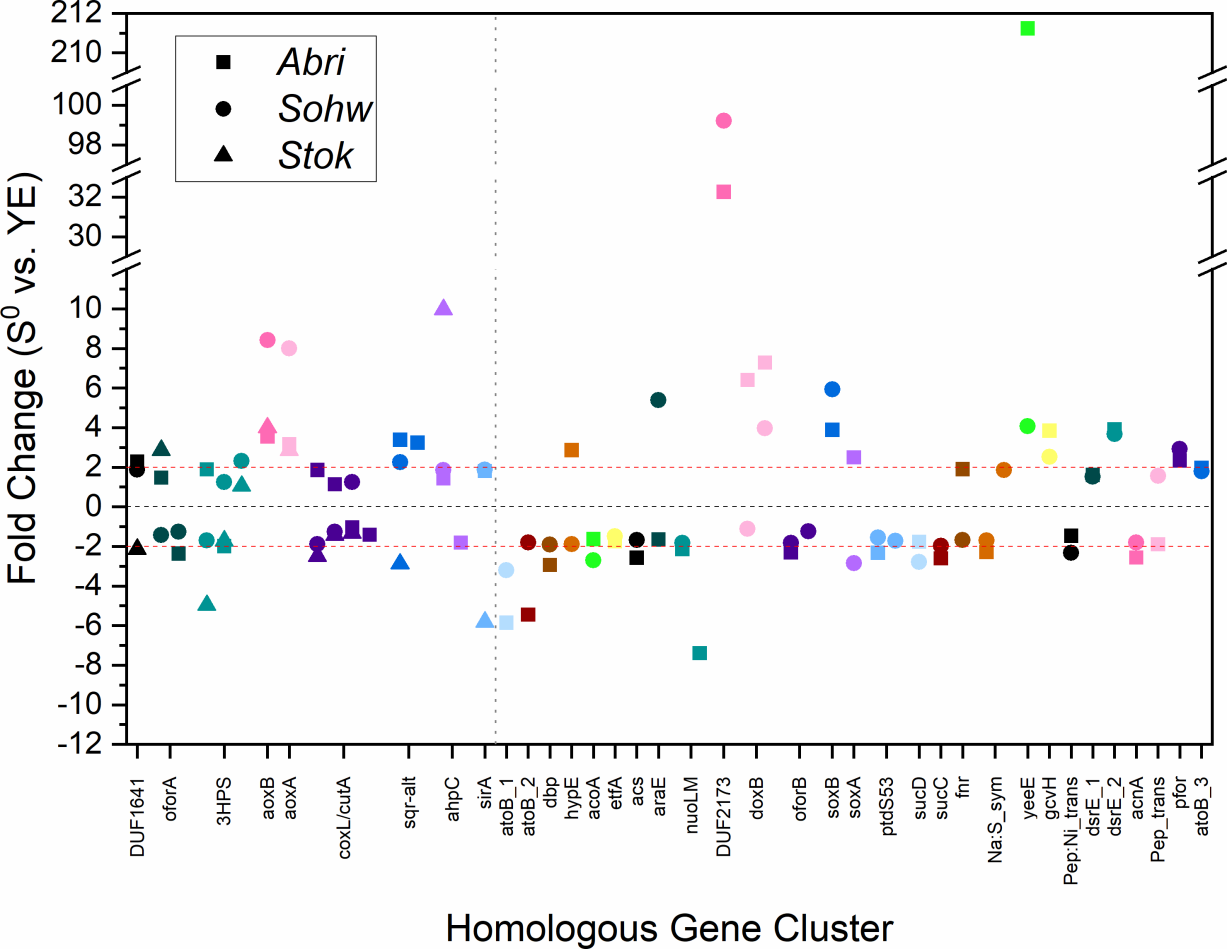
Differential Expression of significant homologous genes from *A. brierleyi*, *S. ohwakuensis*, and *S. tokodaii* (left of vertical dashed line) or *A. brierleyi* and *S. ohwakuensis* (right of vertical dashed line). Red lines indicate a fold-change cut-off of 2.0. Genes are considered noteworthy if all homologous genes are differentially expressed greater than two-fold in each organism. Coloring of the data points distinguishes between homologous gene clusters. *Abbreviations: oforA*/*oforB* (2-oxoacid:ferredoxin oxidoreductase), *3HPS* (3-hydroxypropionyl-CoA synthetase), *aoxA*/*aoxB* (arsenite oxidase), *coxL*/*cutA* (aerobic carbon monoxide dehydrogenase/glyceraldehyde dehydrogenase), *sqr-alt* (putative sulfide:quinone oxidoreductase), *aphC* (peroxiredoxin), *sirA* (response regulator), *atoB* (acetyl-CoA C-acetyltransferase), *dbp* (DNA-binding protein), *hypE* (hydrogenase formation subunit), *acoA* (acetoin:2,6-dichlorophenolindophenol oxidoreductase), *etfA* (electron transfer flavoprotein subunit), *acs* (acetyl-CoA synthetase), *araE* (arabinose:H + symporter), *nuoLM* (NADH:quinone dehydrogenase subunit), *doxB* (terminal oxidase subunit), *soxA*/*soxB* (proton-pumping terminal oxidase subunit), *ptdS53* (peptidase S53), *sucC*/*sucD* (succinate—CoA ligase subunit), *fnr* (ferredoxin:NADP+ reductase), *Na:S_sym* (sodium:solute symporter), *yeeE* (thiosulfate importer), *gcvH* (glycine cleavage system protein), *Pep:Ni_trans* (peptide:nickel transport system), *dsrE* (putative disulfide reductase), *acnA* (aconitate hydratase), *Pep_trans* (peptide transporter), and *pfor* (pyruvate:ferredoxin oxidoreductase).

Principal component analysis (PCA) of the transcriptomic response of the individual biological replicates for each species revealed that the first principal component dimension largely described the variation related to growth in the presence of elemental sulfur (Fig. 6). Genes with a significant differential expression that also correlated strongly (a correlation coefficient >0.5) with the first principal component dimension are likely involved in sulfur oxidation metabolism, including some genes beyond those known to be related to sulfur oxidation. Combining the four species’ transcriptomic profiles revealed 265 homologous gene clusters that satisfied this selection criteria. Only nine of those gene clusters exhibited significant differential expression in all three of the sulfur-oxidizers (*A. brierleyi*, *S. ohwakuensis*, and *S. tokodaii*), and of those nine clusters, only two exhibited a similar direction of regulation in all three species (Fig. 7). Those two gene clusters are annotated as the alpha and beta subunits of a putative arsenite oxidase, which is notably absent in the genome of *S. acidocaldarius*.

A transcriptomic pattern is more apparent when considering only the strong sulfur oxidizers *A. brierleyi* and *S. ohwakuensis*. In this case, 33 genes showed significant differential expression in response to elemental sulfur (Fig. 7). Thirteen of those genes changed in the same direction for both species, of which seven genes are involved in the *hdr* complex. Two genes that were upregulated for both species represent β-subunits of terminal oxidases, indicating the upregulation of ETC components. Among the most highly upregulated genes in these species was a putative membrane protein with a DUF2173 domain (up 99-fold in *A. brierleyi* and 32-fold in *S. ohwakuensis*). This protein exhibits multiple transmembrane domains, indicating an oscillating pattern that may point toward a role in substrate detection and signaling. Notably, this gene was also upregulated 2.3-fold in *S. tokodaii*, although not statistically significant. Another highly sulfur-responsive gene in *A. brierleyi* (up 211-fold) and *S. ohwakuensis* (up 4-fold) was a putative YeeE-type thiosulfate transporter (23), which is conserved in all strong sulfur oxidizers and absent in *S. tokodaii*, *S. acidocaldarius*, and *S. solfataricus*.

### Evolutionary composition of chemolithoautotrophy in the Sulfolobaceae

**Fig 8:**
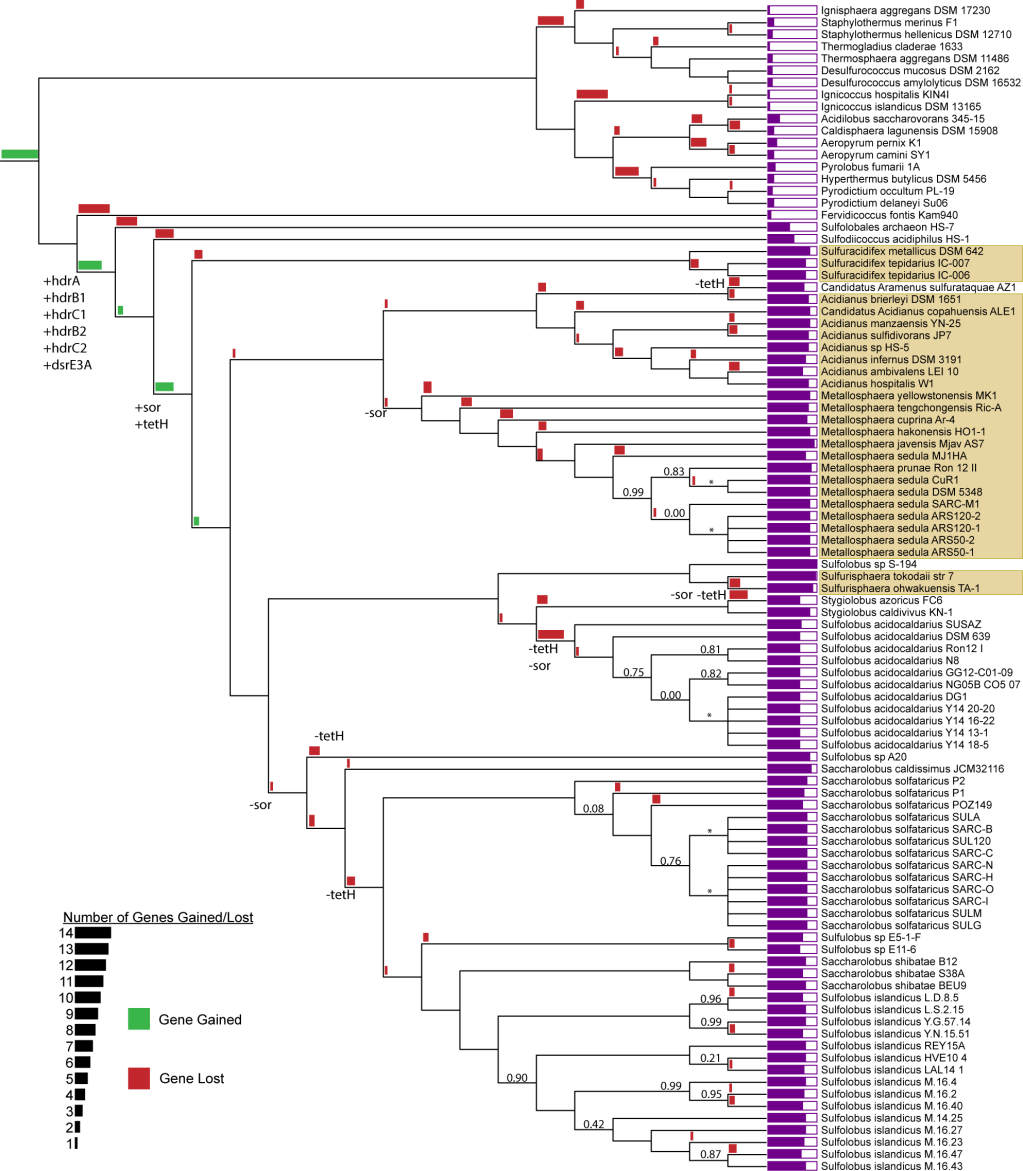
Phylogenetic tree of 485 core single-copy genes of the Sulfolobaceae. Gene gain (green) and loss (red) events of 34 sulfur metabolism genes are represented as bars at each node. Organism names shown in yellow are considered to be capable of sulfur oxidation. Bootstrap values less than 1.00 are indicated for each branch. An asterisk indicates the bootstrap value was undefined. All bootstrap values less than 1.00 occurred at the strain and substrain levels.

Genetic components involved in sulfur oxidation are highly conserved throughout the Sulfolobaceae (Fig. 8). Genes involved in processing persulfide compounds, such as *dsrE3A*, *tusA*, and the *hdr* complex, are present in every member of the family, suggesting that at least some ability to process sulfane sulfur is necessary, at least at some evolutionary point, for thermoacidophilic archaea. Components of sulfur oxidation involved in polysulfide and thiosulfate processing are not ubiquitous but still are more conserved than might be expected. The genes encoding known sulfur oxidation proteins such as SQR, TQO, and SOR largely track with patterns of canonical sulfur oxidation. Surprisingly, the gene most consistently tracking with the sulfur oxidation capability throughout the Sulfolobaceae is *tetH* despite its unclear role in the oxidation of elemental sulfur.

Predicted gene “gain and loss” events relating to sulfur metabolism create a picture of the development of sulfur oxidation capabilities. Acquisition of the highly conserved *hdr* complex, along with electron transport chain-related genes *sqr* and *tqoB*, occurs at the branch point for the Sulfolobaceae, coinciding with adaptation to an acidic environment. The *sor* gene, understood to be a key marker of sulfur oxidation capabilities, is acquired (along with *tetH*) at a later branching point. The sulfur-inhibited *Sulfodiicoccus acidophilus* is excluded from the ancestry of *sor*/*tetH* acquisition, as it branches off from the rest of the Sulfolobaceae prior to this event. In fact, this speciation event coincides with the loss of genes *lbpM1/lbpM2* encoding two lipoate-binding proteins involved in the functionality of the *hdr* complex (14).

Based on this gene acquisition pattern, the ancestral Sulfolobaceae (excluding *S. acidophilus*) were likely strong sulfur oxidizers, developing this phenotype in two stages. The initial acquisition of basal sulfur oxidation capabilities likely occurred with the acquisition of the *hdr* complex and may have been a mechanism to manage RISC toxicity in its sulfur-rich and acidic environment. The ancestor then improved upon this basal level of sulfur oxidation with the acquisition of *sor* and *tetH* to actively facilitate sulfur oxidation for bioenergetic benefit. As species branched off from this initial ancestor and became more heterotrophic, sulfur oxidation capability was sometimes lost. Specifically, the loss of the *tetH* and *sor* genes correlates with the reversion of some Sulfolobaceae lineages to a baseline amount of sulfur oxidation, enough to tolerate their sulfur-rich environments but not enough to support a chemolithoautotrophic lifestyle. One key exception to this trend is the loss of the *sor* gene in the genus *Metallosphaera*. Here, most species are still capable of strong sulfur oxidation despite the absence of *sor*. Thus, the *tetH* gene appears to be the primary marker of strong sulfur oxidation in the Sulfolobaceae.

## DISCUSSION

Engineering *S. acidocaldarius* RK88 to contain the known cytoplasmic components of sulfur oxidation did improve the net sulfate production over its parent strain *S. acidocaldarius* MW001, although not at levels of sulfate generation consistent with strong or moderate sulfur-oxidizing Sulfolobaceae species. This shortcoming in *S. acidocaldarius* RK88 occurs despite interfacing with elemental sulfur in a similar manner to native sulfur-oxidizing species (e.g., *A. brierleyi*, *S. metallicus*, and *S. tokodaii*). However, the observation that these cells tend to form aggregates tethered to the sulfur surface, rather than the cells directly contacting the sulfur, points toward the formation of a soluble sulfur compound that is taken up by the cells. It seems likely that *S. acidocaldarius* RK88 is lacking the genetic components to generate this soluble sulfur compound, thus preventing strong sulfur oxidation. Furthermore, this same limitation in sulfur acquisition may explain the low levels of sulfate generation observed in *S. tokodaii* relative to its high cell density.

**Fig 9:**
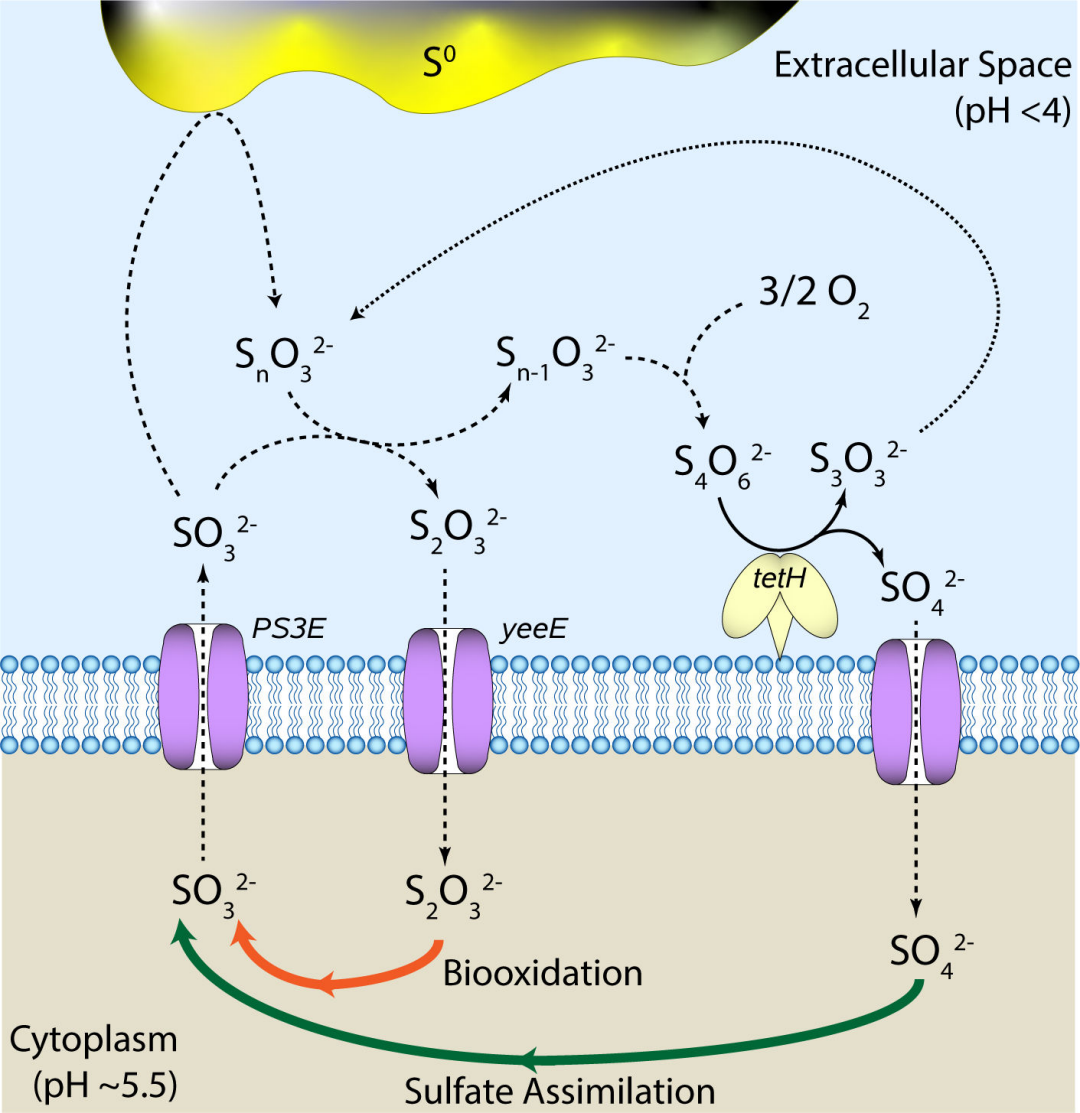
Proposed acquisition mechanism for the active uptake of thiosulfate through sulfur depolymerization by sulfite. The resulting thiosulfate is taken up by the YeeE transporter. Abiotic polythionate byproducts are recycled by tetrathionate hydrolase for further depolymerization. Dashed lines indicate chemical reactions or cross-membrane transport. Solid lines indicate enzymatic reactions (black) or multi-step pathways (orange and green).

The combined genomic and transcriptomic analysis of the sulfur-oxidizing Sulfolobaceae points toward several key genes encoding membrane-bound or extracellular proteins that could facilitate the active uptake of a soluble sulfur compound. First, a putative sulfite exporter, previously identified in *Metallosphaera cuprina* (21), is present exclusively in the sulfur-oxidizing Sulfolobaceae evaluated in this analysis, giving it a phenotype score of 1 (Fig. 2). This fact, combined with the low phenotype score of many sulfite oxidation genes, taken together with the lack of transcriptomic response from these same genes, points toward the Sulfolobaceae exporting sulfite formed during sulfur oxidation rather than biologically oxidizing it to sulfate. Sulfite could then act to initiate the solubilization of elemental sulfur, forming unstable monosulfonate compounds; further attack of these compounds by sulfite could lead to the formation of extracellular thiosulfate (24). This implicates the highly transcribed *yeeE* thiosulfate importer from *A. brierleyi* and *S. ohwakuensis* as the means for the transport of sulfur compounds into the cytoplasm to undergo sulfur oxidation (Fig. 9). In support of this, the *yeeE* gene has a similarly high phenotype score (0.875), and the weak sulfur oxidizer, *S. tokodaii*, is the only sulfur oxidizer lacking this gene.

These RISC species are all thermodynamically unstable in acid (24) and highly reactive with oxygen. Monosulfonates can be quickly converted to polythionates in the presence of oxygen, which have been shown to have some degree of stability in acidic hot springs (25), thereby reducing the pool of available sulfur for thiosulfate formation and uptake. In this case, TetH, which has a phenotype score of 1 and is upregulated on sulfur in *A. brierleyi*, acts in a recycling capacity, hydrolyzing polythionate compounds back into monosulfonates for further degradation to thiosulfate.

This potential mechanism for the active uptake of sulfur involves multiple compounds that are highly reactive in acidic environments and sensitive to oxygen. Thus, the cells maintaining proximity to the solid sulfur substrate would be beneficial by creating a localized high concentration of these sulfur compounds. This improves the rate of sulfur solubilization and reduces the exposure of these compounds to oxygen prior to thiosulfate uptake. Cells aggregating near the sulfur surface, as observed by SEM imaging, would be more effective at sulfur acquisition.

Nanoparticulate cyclic sulfur could also be taken up by the cells through passive diffusion across the cell membrane, taking advantage of the hydrophobic nature of elemental sulfur. However, without a strong presence of sulfide ions to initiate the formation of these nanoparticulates, the process would likely be slow. Recent work demonstrating unexpectedly high stability of sulfide in acidic pH and in the presence of oxygen, likely due to the fully protonated state of sulfide at low pH, emphasizes that the formation of nanoparticulate sulfur would likely be a slow process (26). However, it may explain the base levels of sulfate production observed even in weak sulfur-oxidizing species, such as *S. solfataricus* and *S. acidocaldarius*. Indeed, the idea of passive sulfur acquisition supports the total conservation in the Sulfolobaceae of the HDR complex as a sulfur detoxification mechanism. Only Sulfolobaceae that evolved to acquire sulfur through an active enzymatic process are the ones now capable of leveraging sulfur oxidation for energy conservation and growth.

## MATERIALS AND METHODS

### Strains and cultivation conditions

All Sulfolobaceae species were grown in a low-sulfate formulation of Brock’s Salts (DSM-88 medium) containing: 1.05 g/L (NH_4_)Cl, 0.28 g/L KH_2_PO_4_, 0.25 g/L MgSO_4_·7H_2_O, 0.07 g/L CaCl_2_·2H_2_O, 0.02 g/L FeCl_3_·6H_2_O, 4.5 mg/L Na_2_B_4_O_7_·10H_2_O, 1.8 mg/L MnCl_2_·4H_2_O, 0.22 mg/L Na_2_MoO_4_·2H_2_O, 0.22 mg/L ZnSO_4_·7H_2_O, 0.05 mg/L CuCl_2_·2H_2_O, 0.03 mg/L VOSO_4_·2H_2_O, and 0.01 mg/L CoSO_4_·7H_2_O; media pH-adjusted with concentrated HCl. Cultures were supplemented with 10 g/L of elemental sulfur. Media supplements for specific species included: 2 g/L sucrose and 1 g/L NZ-Amine (*S. acidocaldarius* MW001 and *S. solfataricus* P2); 0.01 g/L uracil (*S. acidocaldarius* MW001); 1 g/L yeast extract (*A. brierleyi* DSM1651, *Metallosphaera hakonensis* HO1-1, *Metallosphaera prunae* RON 12/II, *Metallosphaera sedula* DSM5348, *S. ohwakuensis* TA-1, and *S. tokodaii* str.7); 1 g/L glucose and 1 g/L casamino acids (*S. tokodaii* str. 7); and 0.2 g/L yeast extract (*S. metallicus* DSM6482 and *Sulfuracidifex tepidarius* IC-007). All cultures were 50 mL volumes in 125 mL glass serum bottles vented with foam stoppers; organisms were grown at 65°C (*S. metallicus* and *S. tepidarius*), 70°C (*A. brierleyi*, *M. hakonensis*, *M. prunae*, and *M. sedula*), or 75°C (*S. ohwakuensis*, *S. tokodaii*, *S. solfataricus*, and *S. acidocaldarius* MW001), and pH 2 (*S. metallicus*, *S. tepidarius*, *A. brierleyi*, *M. prunae*, and *M. sedula*) or pH 3 (*M. hakonensis*, *S. ohwakuensis*, *S. tokodaii*, *S. solfataricus*, and *S. acidocaldarius*). Solid media for recombinant *S. acidocaldarius* strains was prepared using a 1:1 mixture of 1.2 wt% phytagel solution and a 2× concentrated solution of the *S. acidocaldarius* media, as described above. Plates used for the selection of plasmid-integrated colonies were prepared by omitting uracil from the solid media, and plates used to select backbone excision from the integrated colonies contained 0.1 g/L 5-fluoroorotic acid.

### Generation of recombinant strains

The *S. acidocaldarius* strain designated RK34 was previously engineered to contain the *sor* (ST1127) gene and the *doxD* and *dox*A genes encoding subunits of TQO (ST1855-1856) from *S. tokodaii* (6). This strain was used as the starting point for further addition of the *sqr* (STK_24850) gene extracted by PCR amplification from *S. tokodaii* genomic DNA. The gene was inserted into *S. acidocaldarius* RK34 directly between *doxDA* genes and the constitutive promoter region directly upstream of these genes, with a *slaB* ribosome-binding site (12 bp upstream of Saci_2354) following the *sqr* gene to facilitate expression of the *doxDA* genes. Flanking regions upstream and downstream of the insertion site were approximately 800 bp and were amplified by PCR from *S. acidocaldarius* RK34 genomic DNA. PCR was performed using Q5 polymerase (New England Biolabs, Inc.); primers are listed in Table 1. These DNA fragments were assembled into a pUC19 plasmid backbone containing the *pyrBEF* cassette (SSO0614-0616) using HiFi DNA Assembly Master Mix (New England Biolabs, Inc.). The resulting plasmid was transformed into chemically competent *Escherichia coli* 5-α and extracted using a Plasmid Miniprep Kit (Zymo Research). The plasmid sequence was verified by Sanger sequencing (Azenta Life Sciences). The extracted plasmid was transformed further into *E. coli* K12 ER1821 (New England Biolabs, Inc.) to undergo methylation.

**TABLE 1:** Primers used for construction of *Saci* RK88 construct

Primer	Function	Sequence
DJW_010S	Linearization of pSVA406 backbone	AGGTCGACCATATGGGAG
DJW_010A	Linearization of pSVA406 backbone	CACTAGTGATATCGAATTCCCG
DJW_016S	Plasmid construct screening	CGATTAAGTTGGGTAACGCC
DJW_020A	Plasmid construct screening	GGGCTTTGTGAGATTAAGACAC
DJW_021S	*Saci* RK34 5’ flanking region	GGCGGCCGCGGGAATTCGATATCACTAGTGCTCTTGAAGCTTTCAGATCG
DJW_021A	*Saci* RK34 5’ flanking region	CTAAAACTTTTGGCATATTTTATCCAAGTAATTCTTCTCTC
DJW_022S	STK_24850 (*sqr*) gene	GAGAAGAATTACTTGGATAAAATATGCCAAAAGTTTTAGTTTTAGGTG
DJW_022A	STK_24850 (*sqr*) gene	CTTCATTGAAGTTTATCTTTGACATTCACCAGCTCGCTAAGAAC
DJW_023S	*Saci* RK34 3’ flanking region	GTTCTTAGCGAGCTGGTGAATGTCAAAGATAAACTTCAATGAAG
DJW_023A	*Saci* RK34 3’ flanking region	GCGTTGGGAGCTCTCCCATATGGTCGACCTGCCATGTTCCATTAGATATTTC
DJW_024S	Plasmid construct sequencing	CGTACTGGAGAGAGTACCTG
DJW_025S	Plasmid construct sequencing	TACTGTATTCTCTCCAGGTGA
DJW_026S	Plasmid construct sequencing	CCTTCAACTGCAAACCAC
DJW_027S	Plasmid construct sequencing	GGGTTTGGGGATTGGATT
DJW_028S	KLD insertion of RBS	ATGTCAAAGATAAACTTCAATGAAGG
DJW_028A	KLD insertion of RBS	ACACATACACCCGATCACCAGCTCGCTAAGAAC
DJW_033S	STK_24850 gene sequencing	GTTGTCTGGGTATTTTAAGAAG
DJW_034A	STK_24850 gene sequencing	GTATGAAACCGCCATCATC
DJW_035A	STK_24850 gene sequencing	CCAACCTTTAACCAATTCAG
DJW_037A	*Saci* RK88 integration screening	CTTCTGTCTCTGAACCATCAGG
DJW_038S	*Saci* RK88 insertion sequencing	CTTACGGAAAGAGAGTTGTTGC
DJW_038A	*Saci* RK88 insertion sequencing	CATTCCATGGTGCTTGAATTATC
TSO-1	Template-switching oligo	GCTAATCATTGCAAGCAGTGGTATCAACGCAGAGTACATrGrGrG
Oligo(dT)VN	Binding to poly-A tail	TTTTTTTTTTTTTTTTTTTTTTTTTTTTTTTTTTTTTTTTVN
S3P	Second-strand synthesis	GCTAATCATTGCAAGCAGTGGTATCAACGCAGAGTACAT

The methylated plasmid was transformed by electroporation into electrocompetent *S. acidocaldarius* RK34 cells and selected for using uracil auxotrophy as previously described (7). Transformed cells were plated on a uracil-free medium and screened for plasmid integration into the chromosome. Colonies with integration were transferred into uracil-containing liquid media to allow excision of the plasmid backbone and then plated on a medium containing uracil and 0.1 g/L 5-fluoroorotic acid to select for removal of the plasmid backbone. Colonies were subsequently screened by PCR to verify removal of the plasmid backbone and retention of the inserted *sqr* region. The positive colony was verified by Sanger sequencing (Azenta Life Sci).

### Pangenome construction and functional annotation

All Sulfolobaceae genomes used for analysis are accessible through the National Center for Biotechnology Information (NCBI) GenBank database or RefSeq database if an equivalent GenBank file is not available (Table 2). All closed genomes in the Sulfolobaceae were utilized, and contig-level assemblies were used in instances where a species did not have a published closed genome. GET_HOMOLOGUES (27) was used for homology-driven clustering of proteins from these genomes using the orthoMCL algorithm (28). Two separate clustering databases were generated: one including the complete set of 79 Sulfolobaceae genomes and one including only the 10 strains analyzed for the extent of sulfur oxidation. These 10 genomes were functionally annotated using BlastKOALA (29), eggNOG-Mapper v2 (30), MicrobeAnnotator (31), signalP v5.0 (32), and TransportDB (33) along with the NCBI annotations from the Genbank files. A consensus annotation for each protein cluster in the 10-genome database was found using an in-house algorithm to reconcile these annotations. The 10-genome and 79-genome protein cluster matrices were mapped to each other using the reference protein for each cluster from the 10-genome database.

**TABLE 2:** List of assemblies used for phylogenetic tree reconstruction

Species and strain designation	NCBI assembly accession number
*Ignisphaera aggregans* DSM 17230	GCA_000145985.1
*Staphylothermus marinus* F1	GCA_000015945.1
*Staphylothermus hellenicus* DSM 12710	GCA_000092465.1
*Thermogladius claderae* 1633	GCA_000264495.1
*Thermosphaera aggregans* DSM 11486	GCA_000092185.1
*Desulfurococcus mucosus* DSM 2162	GCA_000186365.1
*Desulfurococcus amylolyticus* DSM 16532	GCA_000231015.3
*Ignicoccus hospitalis* KIN4I	GCA_000017945.1
*Ignicoccus islandicus* DSM 13165	GCA_001481685.1
*Acidilobus saccharovorans* 345–15	GCA_000144915.1
*Caldisphaera lagunensis* DSM 15908	GCA_000317795.1
*Aeropyrum pernix* K1	GCA_000011125.1
*Aeropyrum camini* SY1	GCA_000591035.1
*Pyrolobus fumarii* 1A	GCA_000223395.1
*Hyperthermus butylicus* DSM 5456	GCA_000015145.1
*Pyrodictium occultum* PL-19	GCA_001462395.1
*Pyrodictium delaneyi* Su06	GCA_001412615.1
*Fervidicoccus fontis* Kam940	GCA_000258425.1
*Sulfolobales archaeon* HS-7	GCA_019704295.1
*S. acidophilus* HS-1	GCA_003967175.1
*S. metallicus* DSM 6482	GCA_009729515.1
*S. tepidarius* IC-007	GCA_008326385.1
*S. tepidarius* IC-006	GCA_008326425.1
*Candidatus Aramenus sulfurataquae* AZ1	GCA_000565255.1
*A. brierleyi* DSM 1651	GCA_003201835.2
*Candidatus Acidianus copahuensis* ALE1	GCA_000632495.1
*Acidianus manzaensis* YN-25	GCA_002116695.1
*Acidianus sulfidivorans* JP7	GCA_003201765.2
*Acidianus* sp. HS-5	GCA_021655615.1
*Acidianus infernus* DSM 3191	GCA_009729545.1
*A. ambivalens* LEI 10	GCA_009729015.1
*Acidianus hospitalis* W1	GCA_000213215.1
*Metallosphaera yellowstonensis* MK1	GCA_000243315.1
*Metallosphaera tengchongensis* Ric-A	GCA_013343295.1
*M. cuprina* Ar-4	GCA_000204925.1
*M. hakonensis* HO1-1	GCA_003201675.2
*Metallosphaera javensis* Mjav AS7	GCA_021654415.1
*M sedula* MJ1HA	GCA_023169565.1
*M. prunae* Ron 12/II	GCA_005222525.1
*M. sedula* CuR1	GCA_000747605.1
*M. sedula* DSM 5348	GCA_000016605.1
*M. sedula* SARC-M1	GCA_001266735.1
*M. sedula* ARS120-2	GCA_001266715.1
*M. sedula* ARS120-1	GCA_001266695.1
*M. sedula* ARS50-2	GCA_001266675.1
*M. sedula* ARS50-1	GCA_001266655.1
*Sulfolobus* sp. S-194	GCA_012222305.1
*S. tokodaii* str. 7	GCA_000011205.1
*S. ohwakuensis* TA-1	GCA_009729055.1
*Stygiolobus azoricus* FC6	GCA_009729035.1
*Stygiolobus caldivivus* KN-1	GCA_009729035.1
*S. acidocaldarius* SUSAZ	GCA_000508305.1
*S. acidocaldarius* DSM 639	GCA_000012285.1
*S. acidocaldarius* Ron 12/I	GCA_000338775.1
*S. acidocaldarius* N8	GCA_000340315.1
*S. acidocaldarius* GG12-C01-09	GCA_001481595.1
*S. acidocaldarius* NG05B_CO5_07	GCA_001481635.1
*S. acidocaldarius* DG1	GCF_002215565.1
*S. acidocaldarius* Y14 20–20	GCF_002215445.1
*S. acidocaldarius* Y14 16–22	GCF_002215485.1
*S. acidocaldarius* Y14 13–1	GCF_002215525.1
*S. acidocaldarius* Y14 18–5	GCF_002215405.1
*Sulfolobus* sp. A20	GCA_001719125.1
*Saccharolobus caldissimus* JCM 32116	GCA_020886315.1
*S. solfataricus* P2	GCA_000007005.1
*S. solfataricus* P1	GCA_900079115.1
*S. solfataricus* POZ149	GCA_015654385.1
*S. solfataricus* SULA	GCA_000968435.2
*S. solfataricus* SARC-B	GCA_000968355.2
*S. solfataricus* SUL120	GCA_003852115.1
*S. solfataricus* SARC-C	GCA_000968395.2
*S. solfataricus* SARC-N	GCA_003852175.1
*S. solfataricus* SARC-H	GCA_003852195.1
*S. solfataricus* SARC-O	GCA_003852095.1
*S. solfataricus* SARC-I	GCA_003852215.1
*S. solfataricus* SULM	GCA_003852155.1
*S. solfataricus* SULG	GCA_003852135.1
*Sulfolobus* sp. E5-1-F	GCA_009601705.1
*Sulfolobus* sp. E11-6	GCA_009602405.1
*Saccharolobus shibatae* B12	GCA_019175345.1
*S. shibatae* S36A	GCA_019175305.1
*S. shibatae* BEU9	GCA_019175325.1
*S. islandicus* L.D.8.5	GCA_000024305.1
*S. islandicus* L.S.2.15	GCA_000022385.1
*S. islandicus* Y.G.57.14	GCA_000022465.1
*S. islandicus* Y.N.15.51	GCA_000022485.1
*S. islandicus* REY15A	GCA_000189555.1
*S. islandicus* HVE10/4	GCA_000189575.1
*S. islandicus* LAL14/1	GCA_000364745.1
*S. islandicus* M.16.4	GCA_000022445.1
*S. islandicus* M.16.2	GCF_000245095.1
*S. islandicus* M.16.40	GCF_000245215.1
*S. islandicus* M.14.25	GCA_000022405.1
*S. islandicus* M.16.27	GCA_000022425.1
*S. islandicus* M.16.23	GCF_000245175.1
*S. islandicus* M.16.47	GCF_000245275.1
*S. islandicus* M.16.43	GCF_000245235.1

### Phylogenetic analysis and inferred tree construction

A phylogenetic tree for the 79 Sulfolobaceae genomes was generated using all 446 core protein clusters that contained no in-paralogs. Protein sequences were aligned using Muscle v5.1 (34), trimmed using trimAL v1.4 (35), and all core alignments were concatenated using MEGA v11.0.13 (36). The concatenated alignment was used to infer a phylogenetic tree for the Sulfolobaceae using FastTree v2.1.11 with the LG + CAT method and 1,000 bootstraps (37) and visualized using the Interactive Tree of Life v5 (38). This phylogenetic tree and the 79-genome pangenome matrix were used as inputs to the Count software (39) to calculate gene gain and loss rates for the whole pangenome and for a specific subset of sulfur metabolism genes, identified using the consensus annotations described above.

### Assessment of sulfur oxidation

Cultures were sampled after 72 h, and optical density was measured by absorbance at 600 nm after a rest period to allow solid sulfur particles to settle. A sample of each culture was collected and measured for sulfate concentration according to the turbidimetric method, as previously described (6). Briefly, 1 mL of culture was spun down at 15,000 × *g* for 5 min to pellet out cell mass and solid sulfur particulates. The supernatant was diluted to a range of 0–10 mM SO_4_^2-^. A solution containing 35 g/L BaCl_2_·2H_2_O, 75 g/L polyethylene glycol MW8000, and 20 mL/L concentrated HCl was prepared fresh prior to each analysis. The solution was activated with 150 µL of 10 mM sodium sulfate for 30 mL of assay solution. One hundred microliter of the diluted samples, along with sodium sulfate standards from 0 to 10 mM, was mixed with 75 µL of the activated assay solution in a clear 96-well plate. Absorbance was measured at 600 nm and converted to sulfate concentration using a linear fit of the standard curve. Each biological replicate was measured in six technical replicates on a single 96-well plate.

### Visualization of cell-sulfur attachment by SEM

Cultures were harvested after 72 h of growth. Forty milliliter of each culture was centrifuged at 2,000 × *g* for 20 min, and the supernatant was decanted. The remaining solids containing cell mass and elemental sulfur were fixed for 1 h using 1 mL of a solution containing 4% paraformaldehyde, 1% glutaraldehyde, and 0.1 M sodium cacodylate buffer. The samples were washed three times in 1 mL of 0.1 M sodium cacodylate buffer, with a 30 s centrifuge step at 2,000 × *g* between each wash. Samples were washed in 1 mL solutions using a series of ethanol concentrations: 70% ethanol, followed by 95% ethanol, followed by two washes in 100% ethanol. The solutions were then dehydrated by critical point drying using Samdri 795 equipment (Tousimis, USA) and sputter-coated with gold and palladium. Coated samples were imaged using a Hitachi SUJ3900 SEM on the same day.

### RNA extraction

Cultures were grown with and without elemental sulfur in the medium to mid-exponential phase before being snap-cooled in a dry-ice ethanol bath. RNA extractions were performed using the NEB Monarch Total RNA Miniprep Kit (New England Biolabs, Inc.), according to the vendor’s directions. One milliliter of culture was centrifuged at 5,000 × *g* for 10 min, avoiding solid elemental sulfur when possible, and resuspended in 1 mL of the provided Lysis Buffer. Extractions were proceeded according to the vendor’s instructions, including the optional DNase treatment. The extracted RNA was eluted in 50 µL of nuclease-free water and stored at −80°C. Total RNA concentration was quantified using a Qubit Fluorometer with the RNA Broad Range Assay Kit (Invitrogen).

### RNA ribodepletion, cDNA synthesis, and sequencing by ONT MinION

For transcriptomic analysis, rRNA was removed from the total RNA samples using an RNase H treatment. Briefly, 10 µg of total RNA was hybridized to species-specific single-stranded (ss)DNA probes (2 µM each) and incubated at 95°C for 5 min with 40 U of murine RNase Inhibitor (New England Biolabs) in 10 mM Tris-HCl, 100 mM NaCl, and 1 mM EDTA, pH 8.0. Thermostable RNase H (31.25 U) and RNase H buffer (New England Biolabs) were added to the hybridized mixture and incubated for 30 min at 50°C. Turbo DNase (7.36 U) and Buffer (Invitrogen) were then added to the mixture and incubated at 37°C for 30 min. Ribodepleted mRNA was extracted from the final mixture using 1.8 volumes of RNAClean XP Beads (Beckman Coulter), washed twice with 70% ethanol, eluted in 16 µL of nuclease-free water, and quantified on a Qubit Fluorometer using the RNA High Sensitivity Assay Kit (Invitrogen).

The resulting mRNA was polyadenylated and reverse transcribed using a modified protocol from Oxford Nanopore Technologies (ONT). Briefly, the mRNA samples were polyadenylated using *E. coli* Poly(A) Polymerase (New England Biolabs, Inc.) followed by a bead clean-up with RNAClean XP beads. Reverse transcription was performed according to the ONT protocol, with the exception that a custom 2 µM (dT)VN oligo primer and 10 µM Template Switching oligo were used (Table 1). Second-strand synthesis similarly used the ONT protocol but with a custom 10 µM second-strand synthesis primer (Table 1), followed by RNA degradation using RNase Cocktail Enzyme Mix (ThermoFisher).

The reverse-transcribed cDNA samples were barcoded and prepared for Nanopore sequencing using the Native Barcoding Kit 24 V14 (Oxford Nanopore Technologies), according to the vendor’s directions. The sequencing libraries for the biological triplicates of the sulfur and non-sulfur conditions were prepared together for each species, resulting in six multiplexed samples per flow cell. Sequencing was performed on a MinION Mk1B with 10.4.1 flow cells using high-accuracy live GPU base-calling with MinKNOW v22.12.7 and Guppy v6.4.6.

### Read processing and transcriptomic analysis

Read trimming was performed by Guppy during base-calling. The resulting reads were filtered using NanoFilt v2.8.0 (40) using Q9 quality and 200 bp length cut-offs. The filtered reads were aligned to coding sequences of the published genomes for each organism (Table 2) using BowTie2 with local alignment (41) and counted using HTSeq (42). Read counts were analyzed for differential expression between the sulfur and non-sulfur conditions using a generalized linear model in EdgeR (43). The library size-adjusted counts per million values for individual genes were used as the input variables for a PCA of the biological replicates within each species. PCA was performed in RStudio using the FactoMineR v2.9 (44). Genes of interest were identified as having a change in differential expression greater than twofold and a correlation coefficient >0.90 for the principal component that most strongly described the split between sulfur and non-sulfur conditions of the biological replicates.

## SUPPLEMENTAL MATERIAL

Data Set S1 mbio.01033-24-s0001.xlsx Pangenome matrices.

Data Set S2 mbio.01033-24-s0002.xlsx Functional annotations.

Data Set S3 mbio.01033-24-s0003.xlsx Transcriptomic data.

Supplemental Material mbio.01033-24-s0004.docx Figure S1 and legends for data sets S1–S3.

ASM does not own the copyrights to Supplemental Material that may be linked to, or accessed through, an article. The authors have granted ASM a non-exclusive, world-wide license to publish the Supplemental Material files. Please contact the corresponding author directly for reuse.
