# Supplementary material for: Phenotype-driven assessment of the ancestral trajectory of sulfur biooxidation in the thermoacidophilic archaea Sulfolobaceae
Source: mBio. 2024 Jul 2;15(8):e01033-24. doi: 10.1128/mbio.01033-24 (PMC11323534; doi:10.1128/mbio.01033-24)
Supplement: Supplemental Material — Figure S1 and legends for data sets S1–S3. [file mbio.01033-24-s0004.docx]

Supplementary Materials for

**Phenotype-Driven Assessment of the Ancestral Trajectory of Sulfur Biooxidation in the Thermoacidophilic Archaea Sulfolobaceae**

Daniel J. Willard *et al*

*Corresponding author. Email: [rmkelly@ncsu.edu](mailto:rmkelly@ncsu.edu)

**This PDF file includes:**

Figs. S1

**Other Supplementary Materials for this manuscript include the following:**

Data S1 to S3

Fig. S1.


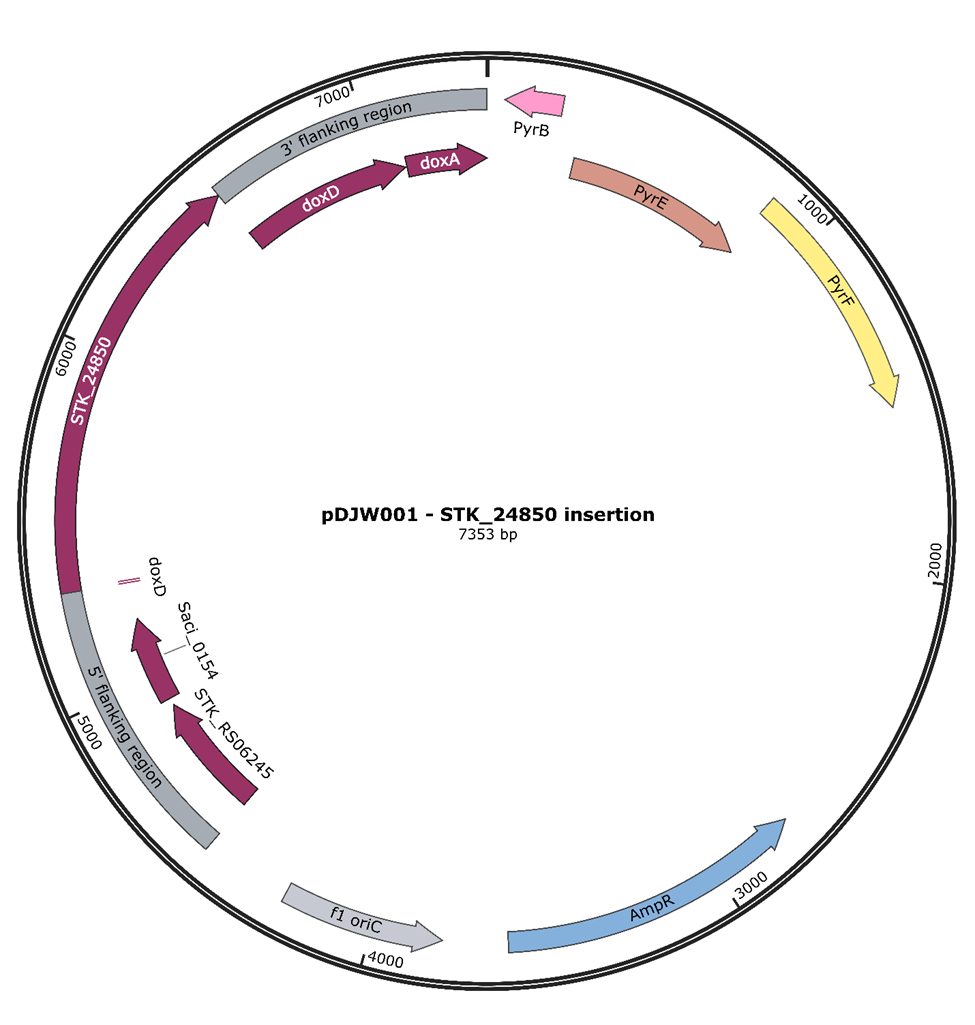


Plasmid map of pDJW001 for insertion of STK_24850 into the *S. acidocaldarius* RK34 strain. The plasmid contains an origin of replication for *E. coli* (labeled “f1 oriC”) and an ampicillin-resistance marker (“AmpR”) for selection of *E. coli* colonies during plasmid assembly. The *pyrBEF* cassette enables colony selection in uracil-auxotrophic *S. acidocaldarius* strains. The gene of interest (STK_24850) has 5’ and 3’ flanking regions on either side of the gene to enable integration of the plasmid into *S. acidocaldarius* RK34.

Data S1. (separate file)

This file contains the pangenome matrices used for homologous protein clustering. The file contains two matrices, each on a separate tab. The tab labeled “Sulfolobales_Pangenome” contains the pangenome matrix for the order-level protein clustering and gene gain/loss analysis. The tab labeled “Exp_Org_Pangenome” contains the pangenome matrix for only the 10 Sulfolobaceae species assessed experimentally for sulfur oxidation capabilities. In both matrices, the first column contains a numeric ID for each cluster followed by an abbreviated functional annotation of the seed protein sequence for that cluster. The remaining columns correspond to each genome evaluated in the matrix and display the protein accession ID(s) from that genome that correspond to each homologous protein cluster.

**Data S2. (separate file)**

This file contains the processed transcriptomic data in response to the presence of elemental sulfur for the four species in this study, with each species on a separate sheet (“Abri_Transcriptomics”: *Acidianus brierleyi*; “Sohw_Transcriptomics”: *Sulfurisphaera ohwakuensis*; “Stok_Transcriptomics”: *Sulfurisphaera tokodaii*; “Saci_Transcriptomics”: *Sulfolobus acidocaldarius*). The first five columns contain accession IDs, genome position, and locus tag for each gene with detectable reads. The “logFC” column indicates log2(fold-change), where a positive value indicates upregulation in the presence of elemental sulfur and a negative value indicates downregulation in the presence of elemental sulfur. The “logCPM” column contains log2(counts-per-million) data for each gene. This value is an aggregate of the two conditions (with and without elemental sulfur) and the biological triplicates run at each condition. Significant regulatory change was determined by the EdgeR software using an F-test, with the resulting F-score for each gene in the column labeled “F”, followed by the associated p-value of the fold-change in the column labled “P-Value”. The column labeled “Significant” indicates whether the F-score for that gene passed the F-test, with a 0 indicating it did not pass, a 1 indicating a pass for upregulation on sulfur, and a -1 indicating a pass for downregulation on sulfur. Finally, the “fold-change” column contains the fold-change value calculated for each gene in non-logarithmic format, with a positive value indicating upregulation on sulfur and a negative value indicating downregulation on sulfur.

**Data S3. (separate file)**

This file contains the extended functional annotation data for the four species used in the transcriptomic analysis of this study. Each species is represented on a separate sheet, with similar nomenclature to the **Data S2** file. The annotation tools are indicated in the column headers. These annotations were used to inform and identify novel genes that were sulfur-responsive for these organisms.
